# Activated carbon derived from sugarcane and modified with natural zeolite for efficient adsorption of methylene blue dye: experimentally and theoretically approaches

**DOI:** 10.1038/s41598-022-22421-8

**Published:** 2022-10-27

**Authors:** Fatma Mohamed, Mohamed Shaban, Shimaa Kotb Zaki, Maysaa Sayed Abd-Elsamie, Radwa Sayed, Mohamed Zayed, Nermein Khalid, Sara Saad, Sara Omar, Ashour M. Ahmed, Abanoub Gerges, H. R. Abd El-Mageed, N. K. Soliman

**Affiliations:** 1 Materials Science Research Laboratory, Chemistry Department, Faculty of Science, Beni-Suef University, Beni-Suef, Egypt; 2 Nanophotonics and Applications Lab, Physics Department, Faculty of Science, Beni-Suef University, Beni-Suef, 62514 Egypt; 3 Department of Physics, Faculty of Science, Islamic University of Madinah, 42351, P. O. Box: 170, AlMadinah Almonawara, Saudi Arabia; 4 Physics Department, College of Science, Imam Mohammad Ibn Saud Islamic University (IMSIU), Riyadh, 11623 Saudi Arabia; 5 Faculty of Science, Micro-Analysis and Environmental Research and Community Services Center, Beni-Suef University, Beni Suef, Egypt; 6 Basic Science Department, Faculty of Oral and Dental Medicine, Nahda University Beni-Suef (NUB), Beni Suef, 11787 Egypt

**Keywords:** Environmental sciences, Chemistry, Nanoscience and technology

## Abstract

The introduction of activated carbon/natural zeolite (AC/NZ) as an efficient and reliable nanoadsorbent for enhancing methylene blue (MB) dye adsorption. By calcining sugarcane waste at various temperatures between 500 and 900 °C, activated carbons (ACs) are formed. Both XRD and SEM were used for the characterization of the prepared adsorbents. Adsorption measurements for the removal of MB dye were made on the impact of pH, beginning MB concentration, and contact time. The maximum AC500/NZ adsorption capacity for MB dye at 25 °C, pH 7, and an AC500/NZ mass of 50 mg was found to be approximately 51 mg/g at an initial concentration of 30 ppm. The pseudo-second-order kinetics model and the Temkin isotherm model describe the adsorption process. The Temkin model shows that the adsorption energy is 1.0 kcal/mol, indicating that the MB-to-AC500/NZ adsorption process occurs physically. Our Monte Carlo (MC) simulation studies supported our findings and showed that the Van der Waals dispersion force was responsible for the MB molecule's physical adsorption. The AC500/NZ adsorbent is thought to be a strong contender for water remediation.

## Introduction

Due to rapid industrialization, massive amounts of harmful waste are dumped into the lake each year. Water contamination is considered to be a major threat to humans and other life forms. The organic dyes are released from the textile, printing, food, and leather industries into the water^1^. These dyes can cause dangerous health problems such as cancer, dermatitis, and allergy^2,3^. Since the dyes are not naturally biodegradable, it is crucial to remove them from wastewater. One of the common dyes, methylene blue (MB), is widely utilized in crucial applications in the food, textile, cosmetic, and pharmaceutical industries. The presence of MB in water increases the oxygen demand which in turn affects aquatic animals. Many techniques have been involved in dye removals such as adsorption, photolysis, photo-Fenton degradation, and photocatalysis as well^2,4–9^. Unfortunately, most of these techniques have significant drawbacks such as high operational cost, long time, low efficiency, sludge production, and formation of secondary pollutants. Among these techniques, the adsorption process is superior to the other techniques due to its high removal efficiency, ease of design, minimum waste production, and low energy requirement^10,11^. Also, it can be applied in treating the dyes in highly concentrated solutions. Developing cost-effective and eco-friendly with high efficiently heterogeneous catalysts is the principal challenge for commercial applications on a large scale^12^.

Activated carbon (AC) is a carbon material like graphite with an irregular and imperfect arrangement structure of microcrystalline carbon. The activated carbon has a porous structure which increases the surface area and decreases density. AC is one of the best adsorbents for removing trace contaminants from air, soil, and water due to its strong physical adsorption^13^. This results from the advantages of the AC such as porous properties, high chemical/thermal stability, unique surface area, surface functional groups, and physicochemical nature of AC^14^. Activated carbons are prepared through physical or chemical activation methods. The physical activation has been reported as more beneficial due to its larger surface area, higher yields, and highly developed porous structure^15^. The activated carbons can be produced from different agricultural wastes such as sugarcane, peat, lignite, wood, and coconut shells. The sugarcane bagasse (SCB) is represented as excellent biomass for AC synthesis due to its availability and low cost. The sugarcane bagasse is produced from the industries of bioethanol, sugar, polyethylene, and ethanol^16^. The composition of SCB is about lignin (20–25%), hemicelluloses (25–30%), and cellulose (40–50%). The disposal of large amounts of sugarcane bagasse wastes has become big environmental pollution, and, consequently, a health hazard in that region. As a result, converting SCB into AC lessens agricultural waste while producing a useful adsorbent at a reasonable price^17^.

There have been numerous experiments done in the past that used AC as an adsorbent to clean up water. For the purpose of methylene blue adsorption in the dark, Amdeha et al. produced AC/TiO_2_^18^. However, the adsorption (%) of MB reached 70% over a period of 60 min. Kuang et al. fabricated AC modified by three surfactants as an adsorbent for methylene blue removal^19^. The adsorption rate was 100% after 120 min at pH 12. However, activated carbon presents several disadvantages such as being expensive, time-consuming, and adsorbent dyes capacity decreases as the number of cycles increases^11^.

Recently, natural zeolite (NZ) is one of the most desirable microporous materials due to its chemical and physical properties^20^. Zeolite is an aluminosilicate mineral that contains alkali and alkaline-earth elements arranged in tetrahedral shape with porous framework structures. It has a high specific surface area, good stability, and tunable hydrophilic/hydrophobic properties. Besides, zeolite is low-cost, abundant, and biocompatibility. Zeolite has attracted properties such as high ion exchange, excellent molecular sieving, and good proton conductivity^21^. These properties have been largely applied in many fields including environmental protection and agriculture. It has been used in several studies to remove different heavy metals^22,23^. However, previous studies showed low efficiency for the removal of dyes due to low sorption capacities^24,25^.

The purpose of this work is to develop activated carbon/natural zeolite (AC/NZ) nanocomposite as an effective and dependable nanoadsorbent for methylene blue (MB) dye from wastewater. Active carbon may be produced at low cost by recycling wastes like sugarcane waste. To lower processing costs and enhance the structures and morphologies of the produced activated carbons (ACs), the calcination temperature of these materials must be optimized. Understanding the mechanisms, kinetics, and isotherms of the adsorption reaction is very critical. It's also necessary to look into how various environmental variables, such as pH, initial MB concentration, and contact time, affect adsorption processes. Finally, it's crucial to use Monte Carlo (MC) simulation to verify the MB's adsorption behavior on the surface of the improved nanoadsorbent.

Based on the above discussion, the combination of the properties of activated carbon with natural zeolite is supposed to be a better composite for dye removal and water treatment. Here, activated carbon/ natural zeolite (AC/NZ) nanocomposite was synthesized and applied for the adsorption of MB dye with high efficiency. The prepared materials are characterized by different techniques including Fourier-transform infrared spectroscopy (FTIR), X-ray diffractometer (XRD), Scanning electron microscopy (SEM) and energy-dispersive X-ray spectroscopy **(**EDX). Also, contact time, initial dye concentration, pH, kinetics, stability, and mechanisms of the adsorption process are studied. The carbonization temperature was optimized during the production of ACs from 500 to 900 °C for the first time. The rule of using the zeolite as a host for improving the adsorbent properties of the optimized sample was studied. The dye removal % increases from 88.6% in the case of AC500 to 99.2% in the case of AC500/NZ in approximately 45 min. Moreover, Monte Carlo (MC) simulation was carried out to verify the MB's adsorption behavior.

## Experimental and computational details

### Preparation of the zeolite, carbon, and AC/NZ

From a raw zeolite mine in Taiz city, natural zeolite was obtained (southwestern the Republic of Yemen). About 10 gm of NZ mine was crushed into small pieces and washed with distilled water several times. Then, it was dried in air and triggered mechanically by ball milling (MTI Corporation, model MSK-SFM) at 4000 rpm for 6 h with a tungsten ring mill. The activated carbon was produced in a laboratory using residual sugarcane as the main biomass source. The SCB ash was acquired from a neighborhood sugar cane juice shop in Beni-Suef, Egypt, and it had a length of 10–25 cm. The SCB raw sample was mixed with sulfuric acid and distilled water for 4 h. The bagasse was then thoroughly washed with hot distilled water and ethanol before being allowed to air dry. In an oven set to roughly 300 °C for 4 h, the SCB was dried. It was ball milled for 5 h at 4000 rpm to a tiny particle size after drying. Then, 40 gm of the resulting SCB were placed in a muffle furnace (Thermo Scientific Thermolyne Model F6010) and heated for 2 h at 500, 700, and 900 °C under N_2_ environment. It was labelled as carbonized carbon AC500, AC700, and AC900, respectively. The bagasse sample allowed cooling back to room temperature. Then, the solid resultant was washed with distilled water several times to remove any impurities and subsequently dried in the air.

**Figure 1:**
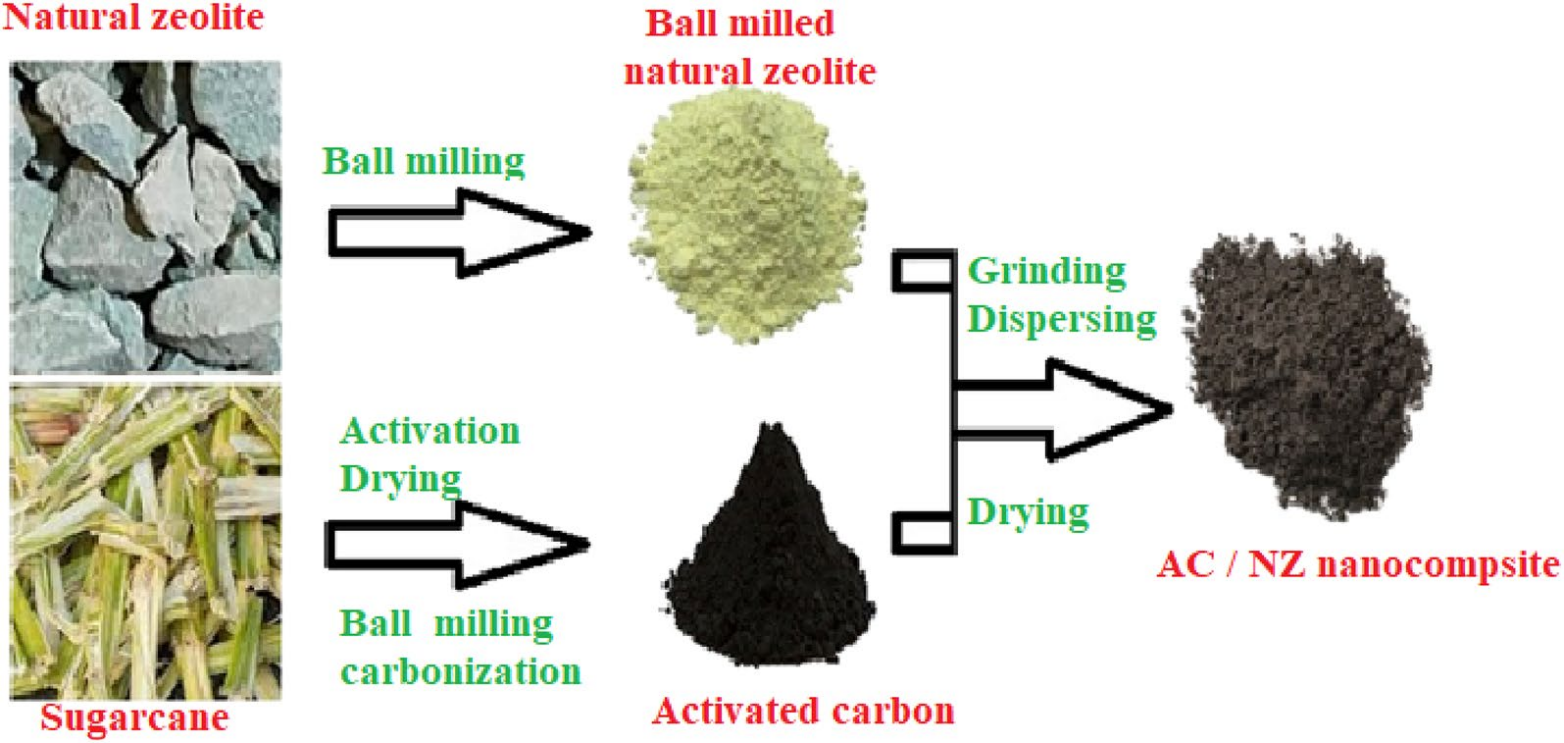
Schematic of the synthesis steps of AC/NZ.

For the manufacture of the AC/NZ nanocomposite, a mortar was used to thoroughly grind 3 gm of the AC500 with 3 gm of the NZ (mass ratio: 1:1) to create a fine powder. The mixture was ground and then dispersed in 100 mL deionized water for two hours at 60 °C in an ultrasonic cleaner (Fisher Scientific model FS110D). The precipitation powder that was produced after filtering was then gathered and baked to dry. The fabrication process for the AC/NZ nanocomposite is depicted schematically in Fig. 1.

### Samples characterization

The X-ray diffraction (XRD; D8 Bruker) analysis was employed at 40 kV and 40 mA with a Cu-Kα excitation source to identify the crystallography of the prepared samples. A scanning electron microscope (SEM; ZEISS EVO) was used to discover the morphology of samples. Fourier transform infrared (FT-IR; Vertex 70) spectra of the fabricated samples were examined to identify the functional groups of the surface. The optical properties of the nanopowders were used at room temperature in the range of 250–900 nm by UV–Vis double beam spectrophotometer (Perkin Elmer Lamba 990).

### Adsorption study

#### Adsorption experiments

The dye solution for methylene blue (C.I. No. 52030; chemical formula: C_16_H_18_N_3_OS) was synthesized in this work at various doses. The adsorption studies were conducted in a dark room at a temperature of 25 °C during the winter of 2022 in Beni-Suef City, Egypt. Initial dye solutions' pH values were monitored using a pH meter and regulated by 0.1 M HCl and 0.1 M NaOH (Jenway 3520). All MB adsorption tests have been carried out in batch mode scale under various experimental conditions, such as starting MB concentration (from 5 to 30 mg/L of MB solution), adsorption contact time (up to 45 min), and pH (3–10) with continuous stirring. Each test was carried out in 50 mL of MB dye solution with desired initial concentration, reusability, and pH value. After the adsorbent (AC, and AC/NZ) was added to the MB solution, the mixture was magnetic stirred (SciQuip, GyroStir 550H) for 20 s at 200 rpm. The variation in the MB concentration was elucidated from the absorption peak measured by UV/Vis spectrophotometer (Jenway 63065). The MB dye concentration was observed at a wavelength of 664 nm within regular periodic intervals.

The quantity of MB uptake by the synthesized AC/NZ at time t (q_t_ (mg/g)) and at equilibrium (q_e_ (mg/g) and MB dye removal% have been obtained utilizing Eqs. (1), (2) and (3), respectively^26,27^.

1 $${\mathrm{q}}_{\mathrm{t }= \left({\mathrm{C}}_{0 }- {\mathrm{C}}_{\mathrm{t}}\right) \frac{\mathrm{v}}{\mathrm{m}}}$$

2 $${\mathrm{q}}_{\mathrm{e }= \left({\mathrm{C}}_{0 }- {\mathrm{C}}_{\mathrm{e}}\right) \frac{\mathrm{v}}{\mathrm{m}}}$$

3 $$\mathrm{MB \, removal \% }= \frac{({\mathrm{C}}_{0}- {\mathrm{C}}_{\mathrm{t}}) }{{\mathrm{C}}_{0}}\times 100$$

which ${\mathrm{C}}_{\mathrm{t}}$, ${\mathrm{C}}_{\mathrm{e}}$, and ${\mathrm{C}}_{0}$ are the MB’s concentrations in mg/L after time t, at equilibrium, and the start of the adsorption process, respectively. The MB volume (V) is measured in mL and m is the AC/NZ mass in mg. All adsorption measurements were repeated three independent times and average values were presented.

#### Adsorption kinetics

Different adsorption mechanisms and kinetic models are used, such as the intra-particle diffusion kinetic model, simple Elovich, pseudo-first-order and pseudo-second-order kinetic models, to determine the most suitable adsorption mechanism and kinetic model for MB adsorption in AC/NZ adsorbent.

Equations (4) to (7) are used to represent the intra-particle diffusion, simple Elovich, pseudo-first-order and pseudo-second-order kinetic models, respectively^28–31^

4 $${\mathrm{q}}_{\mathrm{t}} = {\mathrm{k}}_{1} {\mathrm{t}}^\frac{1}{2}+\mathrm{I}$$

5 $${\mathrm{q}}_{\mathrm{t}} = \frac{1}{\upbeta }\mathrm{ln\alpha \beta }+\frac{1}{\upbeta }\mathrm{ lnt}$$

6 $$ {\text{ln}}\left( {{\text{q}}_{{\text{e}}} - {\text{q}}_{{\text{t}}} } \right) = {\text{ln}}\left( {{\text{q}}_{{\text{e}}} } \right) - {\text{k}}_{{2}} {\text{t}} $$

7 $$\frac{\mathrm{t}}{{\mathrm{q}}_{\mathrm{t}}} = \frac{1}{{\mathrm{k}}_{3}{\mathrm{ q}}_{\mathrm{e}}^{2}}+\frac{\mathrm{t}}{{\mathrm{q}}_{\mathrm{e}}}$$

where k_1_, k_2_, and ${\mathrm{k}}_{3}$ represent rate constants of the Intra-particle, pseudo-first-order and pseudo-second-order propagation models. I refer to a constant related to the boundary thickness. α and β represent the initial adsorption rate at zero time (mg/min) and the extent of surface coverage (g/mg), respectively.

#### Adsorption isotherms

Various adsorption isotherm models have been applied, such as Langmuir, Freundlich, and Temkin adsorption isotherm, to explain the reaction isotherm of the manufactured AC/NZ adsorbent for the tested MB dye. The three models can be represented by Eqs. (8), (9), and (10), respectively^32–34^:

8 $$\frac{{\mathrm{C}}_{\mathrm{e}}}{{\mathrm{q}}_{\mathrm{e}}}= \frac{1}{{\mathrm{K}}_{\mathrm{L }}{\mathrm{Q}}_{\mathrm{o}}}+\frac{{\mathrm{C}}_{\mathrm{e}}}{{\mathrm{Q}}_{\mathrm{o}}}$$

9 $${\mathrm{logq}}_{\mathrm{e}}=\mathrm{ log}{\mathrm{K}}_{\mathrm{F}}+\frac{1}{\mathrm{n}}\mathrm{ log}{\mathrm{C}}_{\mathrm{e}}$$

10 $${\mathrm{q}}_{\mathrm{e}}=\mathrm{ B ln}{\mathrm{K}}_{\mathrm{T}}+\mathrm{B ln}{\mathrm{C}}_{\mathrm{e}}$$

Here, Q_o_ is the maximum amount of dye removed by AC/NZ adsorbents (mg/g), K_L_, K_F_, and K_T_ represent the constants of Langmuir, Freundlich, and binding constant of Temkin isotherm model, respectively. n is the adsorption density, B (= RT/b) is a constant associated with the adsorbed heat, R is the universal gas constant, and T is the absolute temperature.

The dimensionless separation factor (R_L_) could be used to predict the degree of favorability of the Langmuir isotherm for the equilibrium data based on Eq. (11)^35^:

11 $${\mathrm{R}}_{\mathrm{L}}=\frac{1}{(1 +{\mathrm{ K}}_{\mathrm{L}}{\mathrm{C}}_{\mathrm{max}})}$$

where C_max_ represents the maximum initial MB concentration.

### Computational study

The adsorption of methylene blue dye on the AC/NZ nanocomposite and the desorption sites of methylene blue dye on the AC/NZ nanocomposite surface was studied by Monte Carlo (MC) simulation. The initial structures of AC/NZ nanocomposite were taken from the literature^36^. MC simulation was carried out by adsorption Locator module based on COMPASS force field (condensed-phase optimized molecular potentials for atomistic simulation studies) as a force field and use current in the charges section^37^. The basic principles of MC simulation used in this work have been described by Frenkel and Smit^38^. Also, the MD simulation was carried out in this study. In the MD simulations, the electrostatic and van der Waals terms were treated with Ewald and group-based methods, respectively. The MD was simulated under the NPT ensemble for 1 ns, followed by isothermal-isobaric (NPT) conditions at 1 atm and 300 K for 4 ns, with a time step of 1 fs. The temperature and pressure were controlled by a Nose thermostat and Berendsen barostat, respectively. The velocity Verlet algorithm was used in the integration of the equations of motion^39^. The theoretical background of MD simulation is done according to this study^37^.

## Result and discussion

### Characterization

#### Structures and functional groups

**Figure 2:**
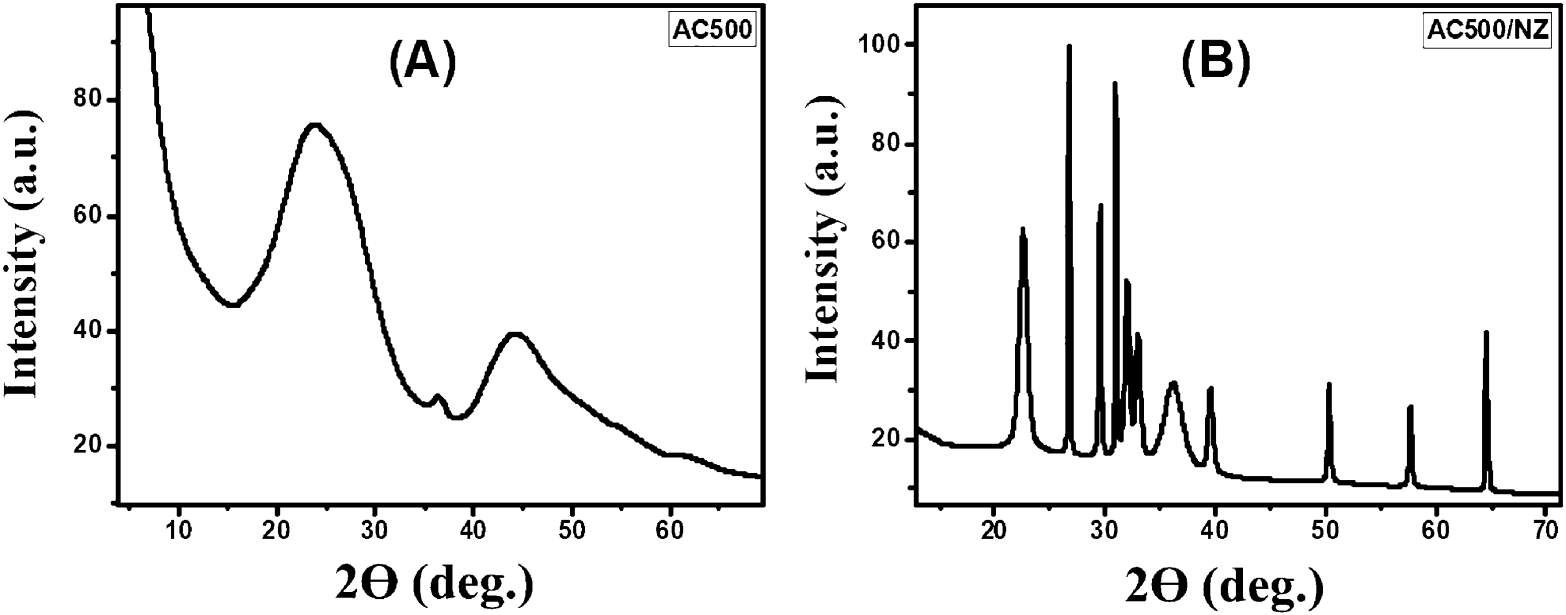
XRD patterns of (**A**) AC500 and (**B**) AC500/NZ.

X-ray diffraction (XRD) examination has been carried out to understand the crystal structure of the prepared samples. XRD charts of AC500 and AC500/NZ in the 2θ range from 0 to 75° are shown in Fig. 2. For the AC500 sample, there are many diffraction peaks located at 2θ of 23.98, 36.63, and 44.38° which confirms the phase of carbonization and activated carbon preparation. The broad bands in XRD patterns characterize the existence of short-range order in the carbon structure which confirms the amorphous phase of carbonization.

The broad diffraction peak observed at 23.98° corresponds to the (002) of the hexagonal carbon-like reflections (JCPDS 50-0926)^40^. The low-intensity peak around 36.63° is attributed (105) of the crystalline graphite (JCPDS 721-616)^40^. The peak located at 44.38° is assigned to the 10 plane (overlapped 100 and 101) of disordered micro-graphite stacking^41^. The graphitic phase may be advantageous for charge transfer since graphitic carbon is a conducting substance. The absence of the peak of cellulose in the XRD pattern confirms the decomposition of cellulose which indicates an amorphous carbon structure with randomly oriented aromatic carbon sheets. The destroyed cellulose chain of sugarcane may improve the exposure of active groups which enhance interaction between AC500 and dye contributing to adsorption^42^.

The AC500/NZ sample showed many XRD diffraction lines. The XRD peaks of zeolite are located at 2θ ~ 26.74°, 29.52°, 31.00°, 31.99°, 32.97°, 39.55°, and 50.28° which confirms the monoclinic phase of zeolite. Based on card JCPDS 00-053-1176, these peaks correspond to Miller indices (− 402), (− 531), (401), (530), (061), (220), and (532), respectively. Natural zeolite's peaks match the structure that has been identified in numerous earlier research^43,44^.

The graphitic phases at 36.63° and 44.38° for AC500/NZ disappeared as a result of the interaction between zeolite and activated carbon during the thermal treatment process, which led to disordered graphitic structure or internal structure. After coupling AC with zeolite, the (002) plane of carbon in the composite experiences a decrease in FWHM and a position shift toward a smaller angle, as shown in Table 1. As a result, the (002) peak's crystallite sizes rose from 24.38 Å for AC nanoparticles to 927.09 for AC/NZ nanocomposite. In addition, the intensities of the diffraction peaks of AC500/NZ nanocomposite became less than the peaks of AC indicating a change in the crystallinity. Also, the microstrain decreased from 6.734 to 0.187% after loading zeolite to AC. The XRD result for AC500/NZ confirms that zeolite has been successfully incorporated into AC500.

**Table 1:** The XRD data for the plane (002) in AC500 and AC500/NZ samples.

Parameters	AC500	AC500/NZ
Pos. (°2Th.)	23.98	22.7425
d-spacing (Å)	3.28	3.33
Crystallite size (Å)	24.38	927.09
Microstrain (%)	6.734	0.187

**Figure 3:**
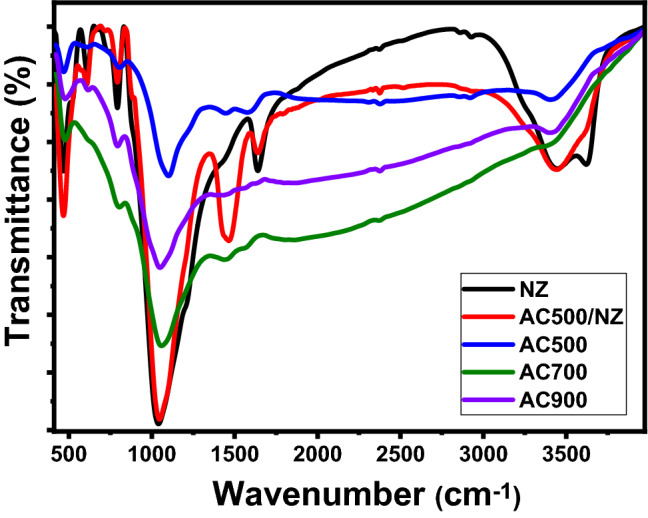
FTIR spectra of NZ, AC, and AC500/NZ.

For dye molecules that have been adsorbed, the functional groups on the surface of nanomaterials may serve as possible attachment sites. The precise functional groups were identified using Fourier transform infrared (FTIR) lattice vibration spectra. To get an infrared spectrum over a broad spectral range with high-resolution spectral data, FTIR is a quick and inexpensive method. The FTIR approach is dependent on how frequently the bonds in an energized molecule vibrate^45–47^. The sample absorbs IR radiation and its molecules are excited into a higher vibrational state when infrared light strikes it. The energy difference between the vibrational modes of an excited and at-rest molecule determines the wavelength of IR radiation that is absorbed by that molecule. The chemical composition and bonding configuration of the materials affect the vibrational transitions. An accurate molecular fingerprint of the material is being created by the ensuing spectrum. Infrared bands can be used to identify the molecular parts and structures of biological and inorganic materials. This makes FTIR useful for a variety of studies. As shown in Fig. 3, FTIR analysis was performed on natural zeolite nanopowders and activated carbon in the 400–4000 cm ^−1^ range.

For AC, the band in the range 3405.48 cm^−1^ is corresponding to the stretch vibration of hydroxyl (O–H)^48^. The bands located at 2920.149 and 2856.403 cm^−1^ indicate present C-H aliphatic stretching and –O–CH_3_ of the aldehyde group, respectively^49^. The bands at 2377.34, 2309.67, and 1576.56 cm^−1^ refer to the C=C stretching vibrations. The band at 1101.00 cm^−1^ indicates the C–O stretching^50^. The bands at 1446.39 and 806.68 cm^−1^ are due to carbonate stretching mode commonly resulting from C–H. The bands at 611.29 and 469.49 cm^−1^ may belong to C–C stretching vibrations.

FTIR of AC500/NZ was shown in Fig. 3. The mixed bands have appeared between AC and zeolite. The bands at 2920.14, 2856.40, 2377.34, 1576.56, 1101.00, and 806.68 cm^–1^ vanishe due to the thermal interaction between AC500 and zeolite. Also, many new bands appear at 1639.47, 1046.25, and 793.04 cm^–1^ in the FTIR spectrum.

The band at 1639.47 cm^−1^ was assigned to the OH bending mode due to adsorbed water on the surface of zeolite^51^. The strongest band at 1046.25 cm^−1^ was assigned to the framework stretching vibration band of Si (Al)-O in the natural zeolite. This indicates the zeolite structure was not destroyed after coupling with AC500. The band at 793.04 cm^−1^ was ascribed to the stretching vibrations of tetrahedral AlO_4_ and SiO_4_ bonds^52^. Additionally, because AC500 is found on zeolite surfaces, the transmittance amplitude of the FT-IR bands for AC500/NZ is lower than in AC500. Furthermore, compared to AC500, the majority of the bands in AC500/NZ were somewhat moved toward a longer wavenumber position.

The stretching vibration of the aromatic ring (C=C), which indicates that the carbonyl group is conjugated with the aromatic ring, was attributed to a strong band at 1580 cm^−1^ in AC500. A slight redshift with temperature increase, to 1565 cm^−1^ for AC700 and AC900, indicated the formation of aromatic ring structure^53^, which is detrimental to the adsorption efficiency of activated carbon as we will show in “Adsorption performance”.

#### Morphologies and elemental composition

**Figure 4:**
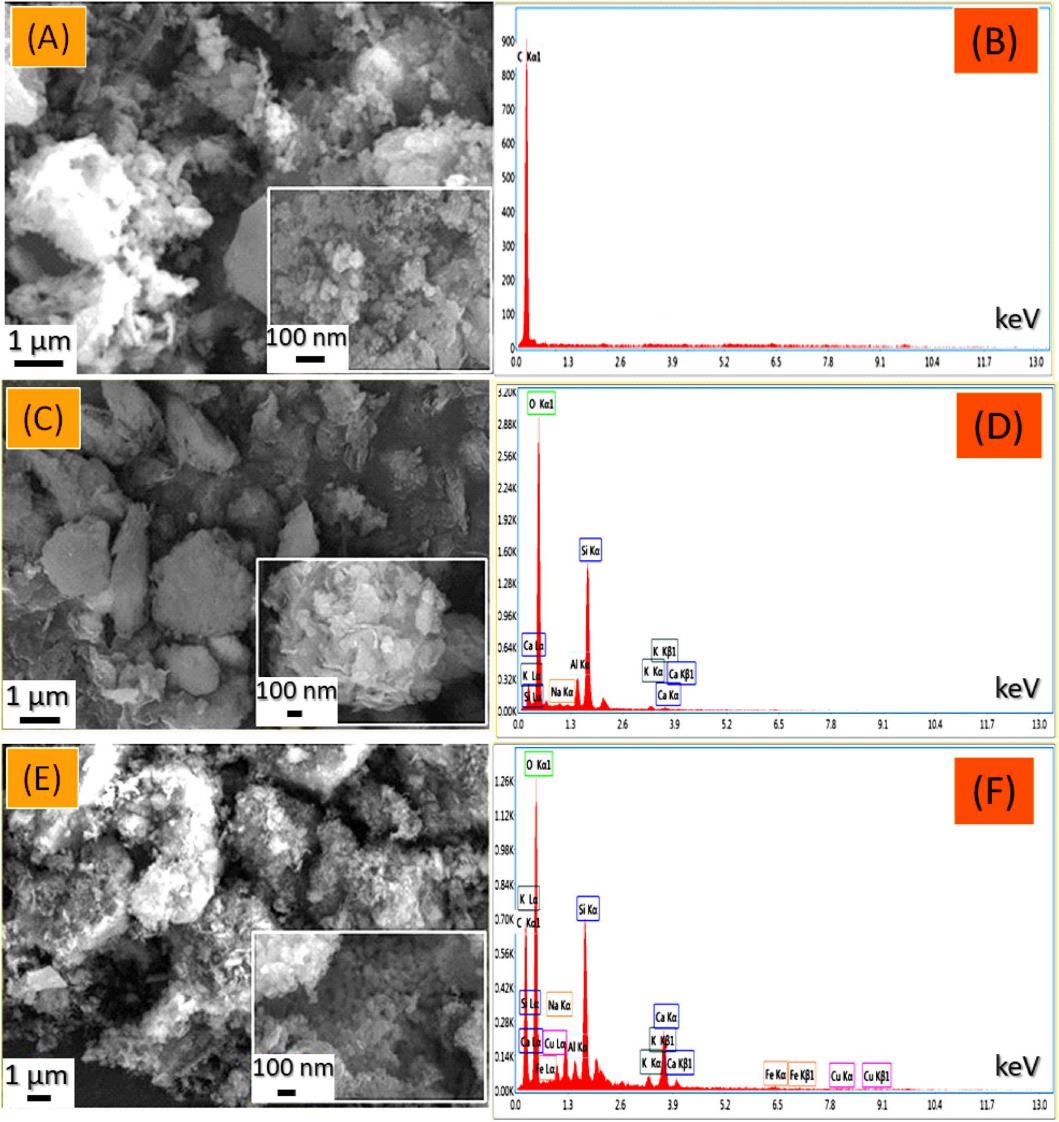
SEM images and EDX elemental analysis of (**A**,**B**) AC500, (**C**,**D**) NZ, and (**E**,**F**) AC500/NZ.

**Figure 5:**
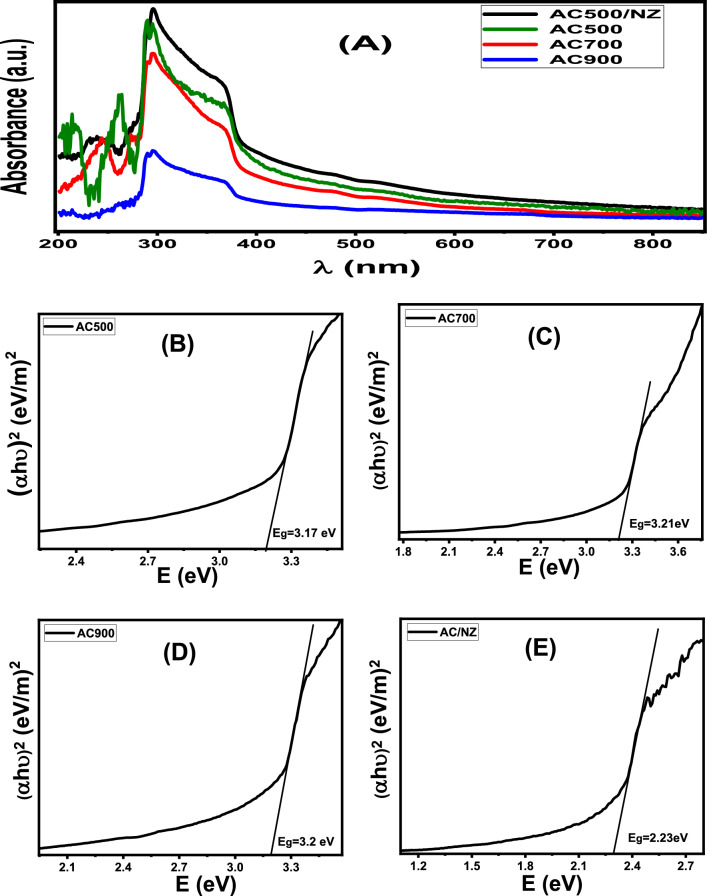
(**A**) Optical absorbance of prepared samples and (**B**–**E**) Plots of (αhν)^2^ versus hν to determine E_g_.

The SEM images and EDX analysis of AC500, NZ, and AC500/NZ adsorbents are illustrated in Fig. 4. The SEM image of AC500, Fig. 4A, shows that AC500 exhibits agglomerated rounded regular shape particles with a less porous surface which consequently affects the surface area for AC500 which in turn affects its adsorption capacity. The EDX analysis (Fig. 4B) performed on the AC500 sample shows the presence of C (100%). SEM image of zeolite, Fig. 5C, illustrated that agglomerated particles, rough surface, different particle sizes, and porous cavities on the surface, while the EDX analysis of zeolite, Fig. 5D, shows the presence of O (65.83%), Si (24.42%), Al (5.91%), Na (1.69%), K (1.30%) and Ca (0.87%). When AC500 is treated with zeolite, Fig. 4E, the SEM image of the nanocomposite shows that the pores in the zeolite surfaces are covered with AC500 particles and converted into agglomerated particles. These images revealed also that zeolite coated the surface of AC and reduced the pores of AC500. The formation of AC500/NZ nanocomposite could be established from alterations in the nanocomposite's morphological topographies when compared to those of AC500 and zeolite. On the other hand, EDX analysis of AC500/NZ, Fig. 4F, shows the presence of C (28.01%), O (50.96%), Si (9.27%), Al (1.55%), Na (2.20%), K (1.26%), and Ca (6.57%) which is considered as another confirmation for the formation of AC500/NZ.

There are many conventional methods for AC/Z synthesis in the laboratory. For example, Prince et al.^54^ synthesized activated carbon /zeolite composite for the removal of heavy metal ions by preparation of activated carbon and subsequent steam-assisted dry gel of tetraethyl orthosilicate and sodium aluminate. Sari et al.^55^ synthesized zeolitic in two steps using extraction of silica from rice husk at 700 °C for 6 h and followed by a hydrothermal process for silica solution in NaOH at 150 °C for 96 h. Mengqi et al.^56^ prepared activated carbon/zeolite composite (CZC) for adsorption and removal of Pb(II) ions from wastewater. They used a two-step trace aqueous-alkali fusion and hydrothermal reaction method using fly ash (FA) as the raw material. Unfortunately, these methods are required high-energy consumption, expensive autoclave, time-consuming, and high cost of precursors.

This indicate our work introduced an inexpensive method for fabricating adsorbents derived from sugarcane waste and natural zeolite stone by ball milling and thermal carbonization techniques. These techniques are very simple, easy, inexpensive, and not required any special equipment. The world production of sugarcane level has reached 60 million tonnes/yearly in about 124 countries. Converting sugarcane waste into valuable products is very beneficial in terms of the economics and environment. The worldwide production of natural zeolite was estimated to be more than 3 million tonnes. The price cost of raw materials for AC/NZ adsorbent is very low. After a survey of many trading companies' websites, the sales price of commercial sugarcane waste and granular natural zeolite is approximately 0.15 and 0.10 US$ per kilogram, respectively. Eventually, the cost of the engineered AC/NZ adsorbent for massive production is estimated to be less than 300 US$ per tonne. Therefore, this adsorbent can be applied industrially applications at a low cost with highly efficient to remove dyes. Also, two issues related to waste management and water pollution can be solved simultaneously by this strategy.

#### Optical absorbance and optical band gap

optical measurement, aqueous suspensions of the nanopowders are used for the UV–Vis absorption studies. About 10 mg of nanopowder sample is dispersed in 50 mL of DI-water under ultrasonic for 120 min. after that, 3 mL of the suspension sample in a cuvette was used for absorbance measurement^3^. The optical absorbance spectra of the prepared AC500, AC700, AC900, and AC500/NZ were measured as shown in Fig. 5.

All nanopowder showed a strong absorption band in the UV range below λ = 350 nm. This band results from an electron jump from the valence band to the conduction band (CB). Then, the absorption decreases rapidly as a function of the wavelength in the visible and NIR region. For AC nanopowders, the absorption intensities overall ranges are decreased with increasing the carbonization temperature from 500 to 900 °C. The AC500/NZ nanocomposite sample has high absorption intensity compared to other nanopowders. This is due to the interaction between AC500 and NZ leading to modification of the electronic structure. Hence, NZ has improved the visible light absorption capability of the AC500 nanopowder. It is observed from Fig. 5 that the absorption edge is shifted lower higher wavelength with increasing the temperature from 500 to 900 °C. As a result, the sample AC500/NZ has a wide absorption band that extends into the visible spectrum. This indicates that this sample can absorb more photons in the visual range. The absorption coefficient (α) was calculated based on measured absorbance (A) by the next equation:

12 $$\mathrm{\alpha }= 2303\mathrm{ A \rho }/\mathrm{ L C}$$

where ρ is the density of nanopowder, L is the length of the quartz cuvette, and C is the concentration of the nanopowder in the suspension.

The optical band gap (Eg) depends on the optical absorption coefficient of nanopowder. The bandgap value can be determined from the Tauc model as follows^57^.

13 $${(\mathrm{\alpha h \nu })}^{2} = {\mathrm{P}}_{0} \left(\mathrm{h \nu }-\mathrm{ Eg}\right)$$

where ${\mathrm{P}}_{0}$ is an independent constant. $\upnu$ is the frequency of the incident photon. h is Planck's constant. In the ${(\mathrm{\alpha h \nu })}^{2}$ − ($\mathrm{h \nu }$) graph, the extrapolating of the straight-line portion is given the value of bandgap ($\mathrm{Eg}$).

From Fig. 5B–E, the bandgap was decreased with the increase of the carbonization temperature of AC. The Eg values were found to be 3.17, 3.20, and 3.23 eV for samples AC500, AC700, and AC900, respectively. The Eg for the AC500/NZ nanopowder was about 2.23 eV as a result of the red-shifted absorption edge. This may be new formation energy levels between AC500 and NZ. Therefore, a combination of AC500 and NZ leads to huge light absorption and reduced bandgap. The narrower bandgap indicates the electrons more easily jump from the VB to the CB under photon irradiation. This suggests that the prepared AC500/NZ can be useful in many solar energy applications.

### Adsorption performance

The stability of the adsorbent for the MB adsorption process was examined, as well as the individual and combined effects of the process variables such as the beginning solution pH, initial dye concentration, and contact time.

#### Effect of carbonization temperature and contact time on the adsorption process

**Figure 6:**
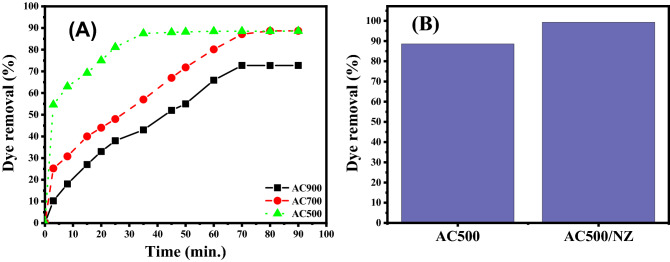
(**A**) The adsorption of 100 mL MB dye solution of concentration 5 mg/L at pH7 using 50 mg AC500, AC700, and AC900 at different times (**B**) The adsorption of 100 mL MB dye solution of concentration 5 mg/L at pH7 using AC500 and AC500/NZ for 45 min.

The use of different carbonization temperatures (500, 700, and 900 °C) had a significant effect on the adsorption behaviour of ACs for MB through the adsorption technique (Fig. 6). The efficiencies of ACs for the removal of MB reached 88.6, 88.6, and 72.7% using 50 mg of AC500, AC700, and AC900, respectively, within 90 min. This finding exhibited that with increasing the carbonization temperature degrees from 500 to 900 °C, the removal of MB decreases. This may be ascribed to the shrinkage and sintering of char at high temperatures (above 500 °C)^58^. Although, elevated temperature favours the formation of pores in carbon, especially when the temperature reaches 500 °C^59^. This is attributed to the use of activators to release tar from the cross-linked framework^60^. However, the intense gasification process damaged a portion of the microporous structure at the higher temperature of 900 °C, which reduced the surface area and pore volume^61^. In addition, elevated temperature increases the percentage of ash and fixed carbon, which reduces volatile matter and solid yields^62^. Therefore, the temperature condition at 500 °C was chosen to be the optimum carbonization temperature for MB adsorption. It can be observed from Fig. 6A that; the dye uptake % was very high during the first stage of the adsorption process, and after that, their increasing rates are reduced to reach the equilibrium state after 30 min in case of AC500 and after 70 min in case of AC700 and AC900. It was observed also that; contact time has no marked effect on the uptake process after reaching the equilibrium using the newly prepared sorbent.

#### Effect of zeolite as a host matrix

After incorporation of zeolite in the carbon matrix, an improvement in adsorption efficiency was observed reaching to be 99.2% after 45 min. The prompt removal rates at the early stage of the reaction are allocated to the existence of a huge surface density of uncovered active spots on the nanoadsorbent's surfaces. By increasing the period of contact between adsorbent and adsorbate, the hot spots are converted to fully occupied sites by MB molecules. As a result, repulsion forces are established between the adsorbed MB molecules on the surface of adsorbents and MB molecules in the bulk liquid phase^63^. Impregnation of zeolite in carbon matrix causes an increase in adsorption efficiency of MB dye as shown in Fig. 6B. This could be related to the negative charge on the adsorbent framework which increases its ability for the adsorption of cationic MB dye^64^. The dye removal % increases from 88.6% in the case of AC500 to 99.2% in the case of AC500/NZ in approximately 45 min.

#### Effect of initial MB concentration

**Figure 7:**
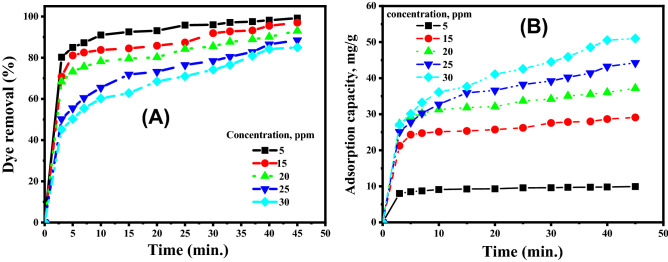
Effect of MB dye concentrations and contact time on (**A**) the removal % of dye and (**B**) The amount of dye adsorbed at 25 °C and pH 7 by 50 mg of AC500/NZ adsorbent.

The variations in the removal % and the amount of MB adsorbed with time using AC500/NZ adsorbent at different initial MB concentrations are shown in Fig. 7A,B, respectively. It can be observed from Fig. 7 that; the adsorption capacity and the dye uptake % were very high during the first stage of the adsorption process, and after that, their increasing rates are reduced to reach the equilibrium state at the end. It was observed also that; contact time has no marked effect on the uptake process after reaching the equilibrium using the newly prepared sorbent. The maximal adsorption percentages for MB dye with initial concentrations of 5, 15, 20, 25 and 30 ppm are 99.2, 97, 93, 88.5 and 85%, respectively, as seen in Fig. 7A. The prompt removal rates at the early stage of the reaction are allocated to the existence of a huge surface density of uncovered active spots on the nano adsorbent's surfaces. By increasing the period of contact between adsorbent and adsorbate, the hot spots are converted to fully occupied sites by MB molecules. As a result, repulsion forces are established between the adsorbed MB molecules on the surface of adsorbents and MB molecules in the bulk liquid phase^63^.

The quantities of adsorbed MB are increased by raising the starting MB concentration as shown in Fig. 7B. This could be ascribed to the increase in the concentration gradient with the elevation of the initial MB concentration. As a result, sufficient draft forces growth has occurred, thereby overcoming the mass transfer resistance between the MB adsorbate and the AC500/NZ adsorbent^65,66^. The maximum adsorption capacities were found to be 9.92, 29.1, 37.2, 44.2 and 51 (mg/g) for MB dye with initial concentrations of 5, 15, 20, 25 and 30 ppm, respectively, at 25 °C, pH 7, and AC500/NZ mass of 50 mg^67,68^.

#### Effect of starting pH

**Figure 8:**
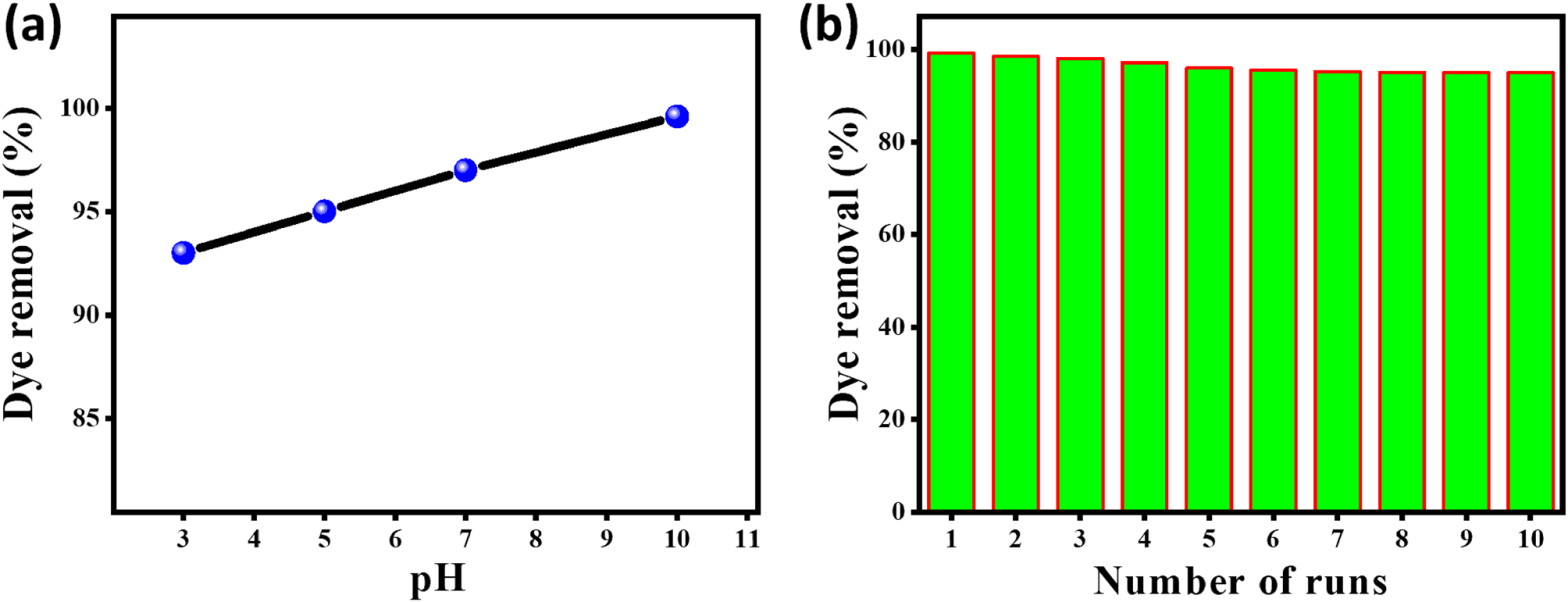
Effects of (**a**) different pH and (**b**) reusability on the adsorption process of 5 mg/L of MB dye on 50 mg of the AC500/NZ adsorbent.

The initial pH of the MB solution can play a significant role in regulating the adsorbent performance due to its impact on the dissociation/ionization of the AC500/NZ adsorbent and their impact on the adsorbent surfaces^69^. So, the electrostatic charges on the AC500/NZ sorbents and the MB sorbate are greatly affected by the pH of the solution. Figure 8a depicts the influence of the starting pH on the removal % of MB dye using AC500/NZ adsorbent. For initial concentrations of 5 mg/L and adsorbent mass of 5 mg per 50 mL of solution, at pH of 3, 5, 7, and 10, the MB dye removal percentage was observed to be 93, 95, 97, and 99.2%, respectively. The lower removal % was obtained at pH 3, which may be attributed to the high mobility of H^+^ ions and the adsorbent surface protonation. Thus, the MB removal% decreases due to the competition between MB molecules and H^+^ ions throughout the adsorption process^70^. But at elevated pH values, pH 5, 7 and 10, the relative elevation in the MB removal% by AC500/NZ is ascribed to the decrease of H^+^ ions concentration^71^.

#### Reusability of the AC500/NZ adsorbent

Figure 8b indicates that the AC500/NZ reusability tests for the removal of MB were repeated ten times using the same adsorbent and dosage. The removal strength of the utilized adsorbent varied substantially across the ten adsorption cycles, according to the findings. The AC500/adsorption NZ's activity decreased after the tenth run, dropping from about 99.2% at the first cycle to 95% at the last. The drop in MB removal% could be attributed to the agglomeration of MB molecules on the surface of the AC500/NZ adsorbent, which shields the adsorbent surface and pores from dissolved MB molecules, resulting in a decrease in adsorption capacity^72^. The result revealed a high performance until 10 consecutive runs with excellent efficiency. So, AC500/NZ is a promising adsorbent for MB dye. The adsorbent reusability determined the commercial availability for industrial applications.

### Adsorption isotherms and kinetics

#### Adsorption isotherms

**Figure 9:**
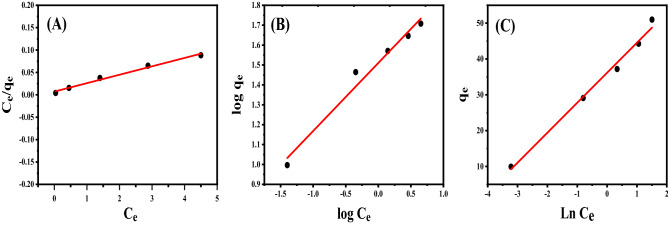
Plots of adsorption isotherms for the adsorption of MB dye by 50 mg of AC500/NZ at 25 °C and initial pH of the solution 7; (**A**) Langmuir isotherms model, (**B**) Freundlich isotherms model and (**C**) Temkin isotherms model.

The statistical significance of the correlation coefficient (R^2^) of Ce/qe & Ce, log(q_e_) & log (C_e_)_,_ and q_e_ & Ln (C_e_) linear fit is a criterion for adjusting the data fitting to Langmuir, Freundlich, and Temkin isotherms, correspondingly. The estimated values of K_T_, K_F_, K_L,_ Q_o_, B, 1/n, and R^2^ were determined from Fig. 9A–C and reported in Table 2. This table shows that MB adsorption on AC500/NZ adsorbent neither follows the Langmuir nor Freundlich isotherm patterns. An important physicochemical property characterizing the interaction of solid surfaces with liquids and gases is the binding energy of adsorbed species. Determination of binding energy is usually performed indirectly by measuring the heat of adsorption. Tempkin isotherm model can be used for this propose by measuring the heat of adsorption ''B values'' according to Tempkin isotherm model. Physical adsorption occurs if the heat of adsorption value is less than 1.0 kcal/mol. Furthermore, with a value of 20–50 kcal/mol, chemisorption occurs. If the heat of adsorption value is between the two (1–20 kcal/mol), both physisorption and chemisorption are involved in the adsorption^73^. The Temkin isotherm model has the largest R^2^ value, so Temkin isothermal model is the model that judges the MB adsorption process. At 25 °C, the R^2^ computed using the Langmuir isotherms was 0.9878, and the RL value for the Langmuir adsorption isotherm is less than one, indicating that MB adsorption is advantageous under the conditions now under investigation^74^. The highest anticipated adsorption capacity of AC500/NZ is 53.4 mg/g, as determined by the Langmuir adsorption isotherm. The Temkin model's B values were less than 1.0 kcal/mol, which suggests that the adsorption process of MB onto AC500/NZ takes place physically at the concentration under investigation^75^.

**Table 2:** Isotherm constants for MB dye adsorption onto 50 mg AC500/NZ at 25 °C and pH 7.

Langmuir isotherm			
Q_o_ (mg/g)	K_L_ (L/mg)	R_L_	R^2^
53.4	2.59	0.013	0.9878

Freundlich isotherm			
**1/n**	K_F_	R^2^	
0.34	32.3	0.9759	

Temkin isotherm			
B (J/mol)	K_T_(L/mole)	R^2^	
8.37	74.6	0.9906	

#### Adsorption kinetics

**Figure 10:**
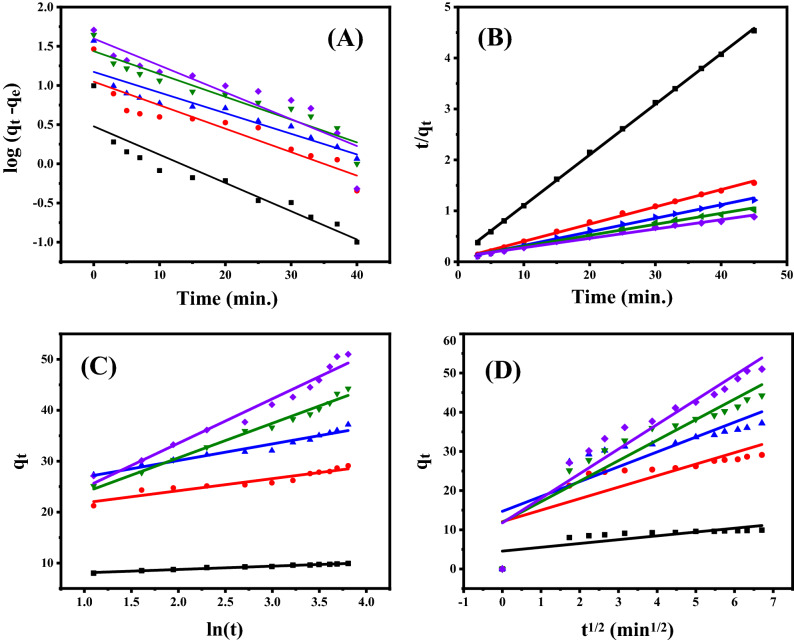
Sorption kinetics of MB dye at 25 °C and pH 7 by 50 mg of adsorbent; (**A**) Pseudo-first order, (**B**) Pseudo-second order, (**C**) Elovich kinetic model and (**D**) Intra-particle sorption.

Under various starting dye concentrations and to examine the most proper adsorption kinetics model, the adsorption of MB dye onto AC500/NZ adsorbent was addressed. Figure 10A–C represent the pseudo-first-order, pseudo-second-order, and Elovich kinetics linear graphs by plotting ln (q_e_ – q_t_) versus t, $\frac{\mathrm{t}}{{\mathrm{q}}_{\mathrm{t}}}$ & t, and q_t_ & ln (t), in order. From the linear plots, the values of the adsorption kinetics parameters such as α, β, q_e_, k_2_ and k_3_, in addition to R^2^ were obtained and presented in Table 3. For all the studied kinetic models, the regression coefficient values in Table 3 confirmed that pseudo-second-order rate law is the kinetic model by which MB adsorption onto AC500/NZ proceeds. The pseudo-second-order kinetic model has the highest correlation coefficient value demonstrating that the adsorption of MB on the AC500/NZ adsorbent almost tracks the pseudo-second-order rate model. The good approximation between the calculated adsorption capacity and experimental adsorption capacity is another proof of this finding. The mechanism of pseudo-secondary adsorption occurs in two steps. Stage of external diffusion during which MB molecules travel from all sides of the fluid to the surface of the outer AC500/NZ. The second stage entails the attachment and adsorption of the adsorbate MB molecules to the surface of the AC500/NZ adsorbent.

**Table 3:** Parameters of the kinetic models for MB dye adsorption onto 50 mg AC500/NZ at 25 °C and pH 7.

Dye concentration (ppm)	5	15	20	25	30
**First-order kinetic model**					
K_2_	0.0829	0.0686	0.0605	0.0667	0.0783
q_e_	1.6	2.8	3.2	4.2	4.9
R^2^	0.8763	0.8392	0.858	0.902	0.8316
q_e_ exp	9.92	29.10	37.20	44.25	51
**Second-order kinetic model**					
K_3_	0.0891	0.0187	0.0126	0.0054	0.0036
q_e_	10.10	29.58	37.73	46.29	54.64
R^2^	0.9996	0.9972	0.9965	0.9939	0.989
q_e_ exp	9.92	29.10	37.20	44.25	51
**Elovich kinetic model**					
β (g/mg)	1.5278	0.4219	0.3076	0.1472	0.1146
α (mg/min)	53,234.23	8506.14	4629.45	83.88	55.05
R^2^	0.9842	0.9156	0.9552	0.9851	0.9697
**Intraparticle diffusion kinetic model**					
K_1_	6.27	5.23	3.7	2.93	0.97
I	11.75	11.94	14.71	12.07	4.55
R^2^	0.8905	0.8452	0.6593	0.6298	0.5706

#### Sorption mechanisms

To comprehend the adsorption kinetics process and rate-controlling steps, the practical data are fitted for Weber's Intra-particle diffusion. Figure 10D represents the linear fit of q_t_ versus t^1/2^. The obtained straight line proposes that the Intra-particle diffusion model is applicable in the study case. The values of K_1_ and I, Table 3, are obtained from the slope and intercept of the linear fitting, respectively. The intercept I ≠ zero signifies that Weber's Intra-particle diffusion model is not the sole rate-scheming route in estimating the adsorption reaction kinetics^76^. The intercept I in Fig. 10D refers to the boundary layer effect. The surface adsorption contribution in the rate-controlling stage increases with increasing intercept value^76^.

The activated carbon, zeolite, and activated carbon/zeolite composite were used to adsorb many harmful materials. In this regard, Xiaoqin et al.^77^ fabricated activated carbon/zeolite (AC/Z) composite using lithium-silicon-powder waste as adsorbent for methylene blue removal with an adsorption capacity of about 15.49 mg/g. Hameed et al.^78^ prepared a mesoporous activated carbon/zeolite (AC/Z) composite by chemically facilitated NaOH activation and hydrothermal treatment with oil palm ash as substrate. The maximum adsorption capacity for removal of MB dye is about 47.95 mg/g with an initial dye concentration of 50 ppm at 30 °C. The maximum adsorption capacity for the removal of MB dye using natural zeolite treated with NaOH, commercial zeolite, and sodium dodecyl sulfate (SDS)-modified zeolites is about 47.3, 22.0, and 5.6 mg/g respectively^20,79,80^. Selhan et al.^81^ obtained activated carbons from waste biomass by sulfuric acid activation with an adsorption capacity of about 16.4 mg/g for MB. Moreover, Hameed et al. prepared^82–86^ many adsorbents such as activated carbon, zeolite/chitosan, and chitosan/sepiolite for the removal of MB dye. Their results showed that the adsorption capacity with an initial dye concentration of 50 ppm at 30 °C was no more than 53.70 mg/g. This value is less than our result which is 51.0 mg/g for AC500/NZ with a low initial concentration of 30 ppm at 25 °C.

### Simulation results

**Figure 11:**
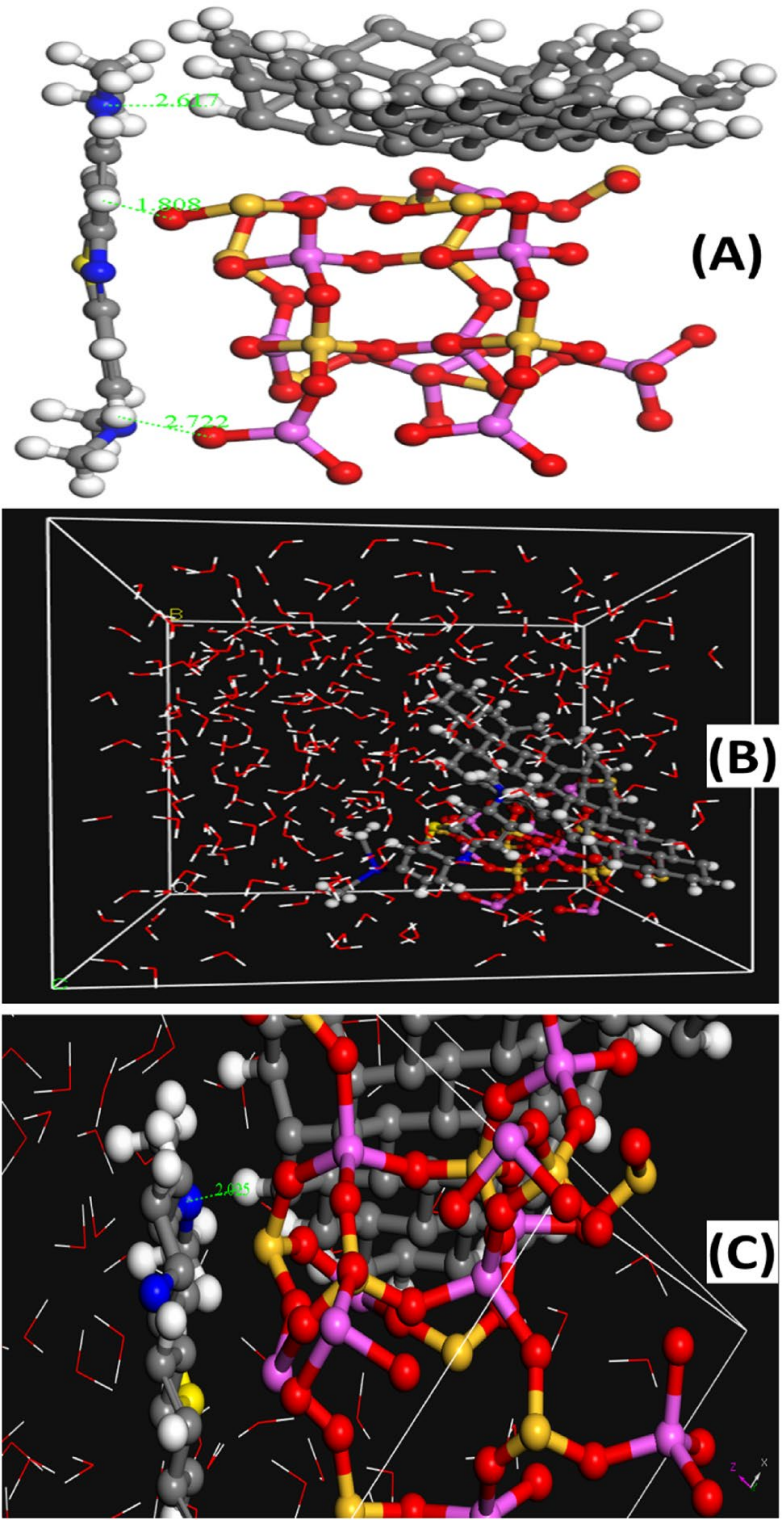
The (**A**) adsorption configurations and (**B**) simulation conformation of the MB dye adsorbed on the AC/NZ nanocomposite surface, obtained from MC simulation; and (**C**) MD snapshots at 5000 ps of the adsorption of methylene blue dye on the AC/NZ nanocomposite surface, the bond length is in Angstroms.

Figure 11A summarizes the lowest configuration that resulted from the adsorption of methylene blue dye on the AC/NZ nanocomposite surface in a dry condition (without a solvent). Table 4 shows the MB dye absorbed on the AC/NZ nanocomposite surface's adsorption (E_ads_), interaction (E_int_), and deformation (E_def_) energies as well as substrate-adsorbate configurations (dE_ads_/dN_i_), in which one of the adsorbate components has been eliminated. Different donor and acceptor sites for hydrogen bonds (HBs) can be found in the MB dye molecule. As a result, the hydrogen atom of the AC/NZ nanocomposite and the nitrogen atom of the methylene blue dye molecule have formed a hydrogen bond at a distance of 2.61 Ǻ. Additionally, as shown in Fig. 11A, the oxygen atom of the AC/NZ nanocomposite has a hydrogen connection with the hydrogen atom of the methylene blue dye molecule at a distance of 1.80 Ǻ. The adsorption of methylene blue dye on the AC/NZ nanocomposite surface is exothermic, energetically advantageous, and spontaneous because of the presence of the intermolecular interactions, according to the results of the Eads methylene blue dye absorbed on the AC/NZ nanocomposite surface is negative. Additionally, it was found that the MB dye was absorbed on the surface of the AC/NZ nanocomposite in a parallel mode, confirming the potent interactions between the methylene blue dye and the atoms of the surface. The adsorption of the MB dye onto the AC/NZ nanocomposite surface may be attributed to the contribution of the electrons of nitrogen and oxygen, according to analysis of the molecular structures of the methylene blue dye and the surface (chemical adsorption). Additionally, the physical adsorption of the methylene blue dye onto the surface of the AC/NZ nanocomposite can be attributed to the Van Der Waals dispersion forces, which support the experimentally obtained findings.

**Table 4:** Adsorption energy (kcal/mol), rigid adsorption energy, deformation energy, and substrate-adsorbate configurations (dE_ads_/d_Ni_) for the adsorption configuration of MB dye on the AC/NZ nanocomposite surface.

Adsorption energy	Rigid adsorption energy	Deformation energy	DR : dEad/dNi
− 70.55	− 20.16	− 70.02	− 700.12

**Figure 12:**
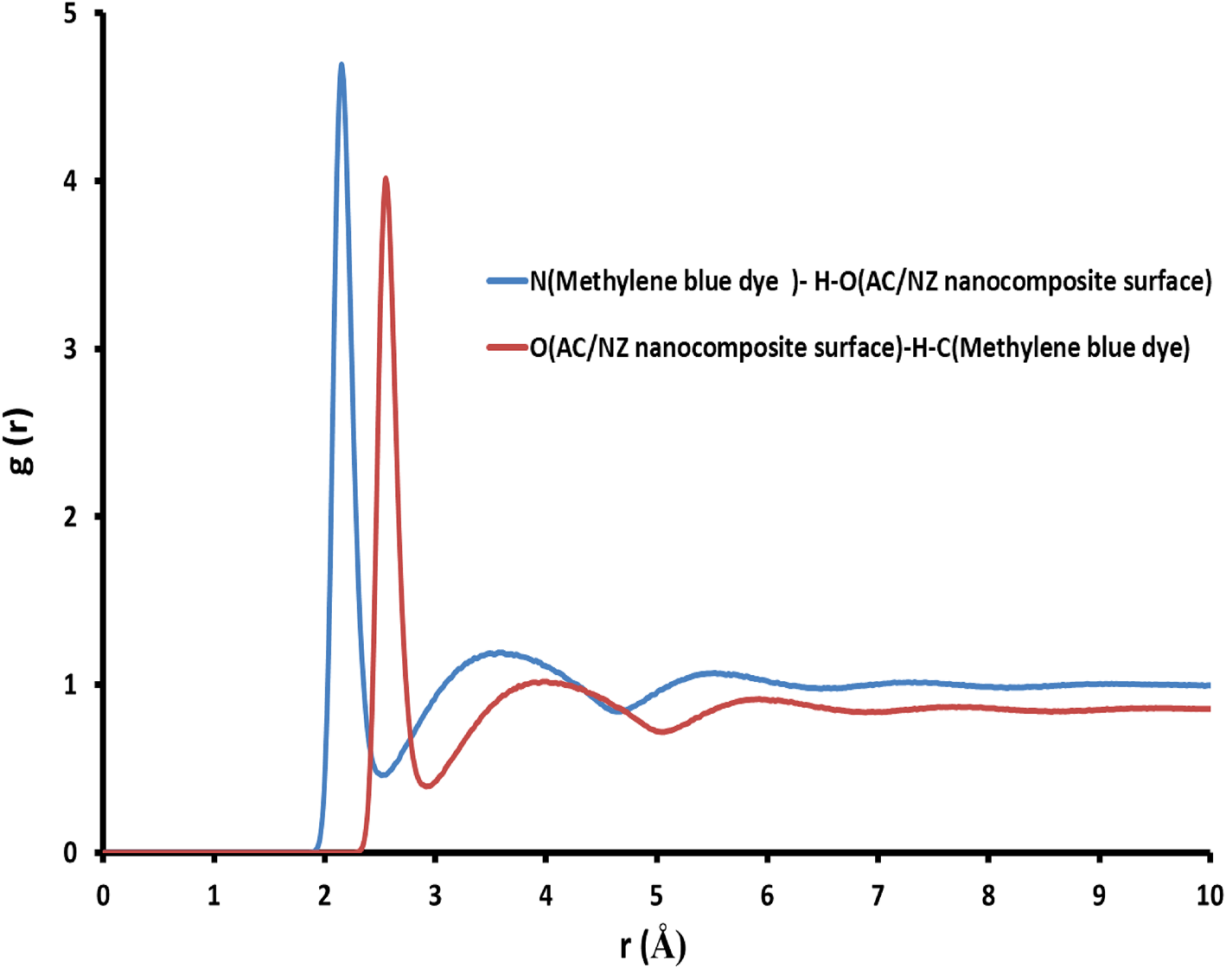
The RDFs for the interaction sites of MB dye with the AC/NZ nanocomposite surface atoms in the presence of water at 5 ns.

The MD simulation was used to investigate the influence of the presence of water solvent molecules on the adsorption of methylene blue dye on the AC/NZ nanocomposite surface, in which the configuration of methylene blue dye absorbed on the AC/NZ nanocomposite surface obtained from MC simulation was simulated in explicit water using MD. Figure 11B depicts the final simulated conformation of methylene blue dye absorbed on the AC/NZ nanocomposite. During the simulations, the aqueous solution's water molecules were free to migrate and interact with the methylene blue dye and the AC/NZ nanocomposite. MD snapshot at 5000 ps of the adsorption of methylene blue dye absorbed on the AC/NZ nanocomposite is shown in Fig. 11B. Since the MB dye has several HB donor and acceptor sites, it has created a number of hydrogen bonds with the atoms in the AC/NZ nanocomposite. Additionally, the hydroxyl hydrogen atoms of the MB dye molecule were used to generate HBs with the oxygen atoms of the AC/NZ nanocomposite. Figure 11C shows that in water, the methylene blue dye molecule established coordination bonds with the atoms of the AC/NZ nanocomposite. Both intramolecular hydrogen bonds (HBs) between the functional groups of the methylene blue dye molecule and hydrogen bonds (HBs) between the methylene blue dye and water molecules have been found in water systems. Methylene blue dye still interacts with the atoms of the AC/NZ nanocomposite even in the presence of water molecules, according to the MD simulation. Radial distribution function (RDF) was computed from the MD simulation to gain more insights into the stability of MB dye-AC/NZ nanocomposite surface complex in water explicitly. This RDF can help us to understand the interaction between MB dye molecule and the AC/NZ nanocomposite surface. RDF is explained as the probability of locating particle “B” within the range (r + dr) of particle A and is usually expressed as g(r). The production of hydrogen bonds with water and the interaction of the methylene blue dye molecule dye with the AC/NZ nanocomposite surface were both investigated using this method. The RDFs between the methylene blue dye molecule and the surface atoms of the AC/NZ nanocomposite are shown in Fig. 12. As observed in Fig. 12, the bonds formed by the MB dye's nitrogen atom and the hydroxyl group on the surface of the AC/NZ nanocomposite (N_(Methylene blue dye)_ − H–O_(AC/NZ nanocomposite surface)_) have a bond length of 2.28 Å. The bonds between the hydrogen atom of the methylene blue dye and the oxygen atom of the AC/NZ nanocomposite surface (O_(AC/NZ nanocomposite surface)_-H-C_(Methylene blue dye)_) have a bond length of 2.55 Å. These two interactions, which took place at high intensities, demonstrated that methylene blue and AC/NZ nanocomposite interact strongly. RDFs show that even in the presence of water molecules, methylene blue dye still interacts with the surface of the AC/NZ nanocomposite.

## Conclusion

In summary, using sugarcane waste as the primary feedstock material allows for the successful preparation of the composite made of zeolite and activated carbon. For MB dye from aqueous solutions, the newly created nanocomposite demonstrated a notable efficiency. The shrinking and sintering of char at high temperatures may be the reason why the carbonization temperature for AC was designed to be 500 °C. The pH, duration, and dye concentrations were just a few of the variables that were tuned for the adsorption process. The best kinetic model for MB adsorption, as determined by the kinetic testing, was pseudo-second-order. The determined isothermic parameter values supported the good interaction between MB dye and AC500/NZ as well as a strong ion-exchange mechanism during MB adsorption. The Temkin model offered the best fit for the isotherms. Finally, the straightforward creation of zeolite-activated carbon composite from sugarcane waste could both achieve the goal of treating waste water with waste sugarcane and offer a workable solution to the problem of the accumulation of these wastes. Analysis of the molecular structures derived from the MC simulation of the MB adsorbed on the AC/NZ nanocomposite surface surfaces indicates that the adsorption may be related to the Van Der Waals dispersion forces, which supports the experimental findings. In the presence of water molecules, MB adsorbs on the surface of the AC/NZ nanocomposite, according to MD modeling.

## Data Availability

The datasets used and analyzed during the current study available from the corresponding author on reasonable request.
